# Short- and long-term outcomes after perioperative EOX therapy versus upfront surgery for gastric cancer: a single-centre propensity score–matched cohort study

**DOI:** 10.1186/s12893-025-02919-4

**Published:** 2025-04-28

**Authors:** Johan Back, Ville Sallinen, Akseli Bonsdorff, Arto Kokkola, Pauli Puolakkainen

**Affiliations:** 1 Department of Abdominal Surgery, Helsinki University Hospital and University of Helsinki, Meilahti Tower Hospital, Building 1, Haartmaninkatu 4, PO Box 340, Helsinki, 00029 HUS Finland; 2 Department of Transplantation and Liver Surgery, Helsinki University Hospital and University of Helsinki, Helsinki, Finland

**Keywords:** Perioperative chemotherapy, EOX, Complications, Gastric cancer

## Abstract

**Introduction:**

Despite radical surgery, gastric cancer (GC) survival rates remain low in Western countries. Randomised trials suggest that perioperative chemotherapy downstages disease, improving long-term survival without increasing complications. We compared outcomes for upfront surgery (US) versus surgery combined with perioperative EOX (epirubicin, oxaliplatin, capecitabine) therapy for short- and long-term survival.

**Methods:**

We analysed 310 patients who underwent curative intent gastrectomy for GC at a single tertiary centre from 2006 to 2017. Patients were assigned to the EOX group (*n* = 105) or the US group (*n* = 205). Propensity score matching (PSM) was utilised to balance baseline characteristics, clinical stage, surgery type, and histology. Short-term outcomes included the Comprehensive Complication Index (CCI) and 30-day mortality, while long-term outcomes were overall survival (OS), disease-specific survival (DSS), and disease-free survival (DFS).

**Results:**

After PSM, 102 patients remained in each group. The EOX group exhibited significantly lower preoperative haemoglobin levels compared to the US group, but other baseline characteristics were comparable. Tumour-related outcomes favoured the EOX group, with significantly smaller tumours (*P* < 0.001), fewer metastatic lymph nodes (*P* = 0.004), and lower tumour stages overall. Splenectomy was more common in the US group (40.2% versus 23.5%, *P* = 0.011). Postoperative complications were similar between groups, although ICU admissions were more frequent in the EOX group (16.7% versus 6.9%, *P* = 0.030). Thirty-day mortality rates were low and comparable (1.0% in the EOX group versus 2.0% in the US group, *P* = 1.000). Long-term outcomes, including overall survival (OS), disease-specific survival (DSS), and disease-free survival (DFS), showed no significant differences between the groups.

**Conclusions:**

Perioperative EOX therapy is as safe as upfront surgery and significantly reduces metastatic lymph nodes and tumour size, suggesting its role in downstaging the disease. However, despite these promising oncological responses, this benefit does not translate into improved long-term survival.

**Supplementary Information:**

The online version contains supplementary material available at 10.1186/s12893-025-02919-4.

## Introduction

### Overview on gastric cancer

Approximately 1 million patients are diagnosed with gastric cancer (GC) annually worldwide, with GC representing the fourth leading cause of cancer-related deaths globally [1]. Due to the late onset of symptoms, GC is usually detected at an advanced stage in Western countries, where screening is not routinely implemented.

### Current standard treatment

To date, gastrectomy with a D2 lymphadenectomy and perioperative chemotherapy are considered the gold standards for locally advanced GC (clinical T2 or beyond with or without nodal involvement) in the West [2]. Despite advances in the treatment of GC, patient prognosis remains poor, with an estimated 5-year survival of 30% in Finland [3]. Adjuvant chemotherapy in Western settings has not significantly enhanced overall survival (OS) rates [4–6].

### Differences in treatment outcomes

By contrast, randomised controlled trials in Japan demonstrated a greater benefit from adjuvant chemotherapy, resulting in it remaining a standard practice in that country [7]. To improve survival, various treatment strategies such as perioperative treatment alongside surgery have evolved.

### European trials

Two European trials shifted the focus towards neoadjuvant or perioperative treatment modalities (in the West). The MAGIC trial randomised 503 patients undergoing curative gastrectomy surgery alone or perioperative (3 cycles before and 3 cycles after surgery) ECF (epirubicin, cisplatin, and 5-fluorouracil) plus surgery. MAGIC showed, firstly, that the resected tumours were less advanced in the perioperative chemotherapy group [8]. Secondly, the postoperative morbidity and mortality were similar between groups. Finally, perioperative chemotherapy improved 5-year OS from 23.0% to 36.3%. These findings were later supported by a similar study, the FFCD ACCORD- 07 study, which randomised 224 patients to receive surgery alone or 2–3 cycles of cisplatin and 5-fluorouracil perioperatively [9].

### Evolution of chemotherapy regimens

The triple therapy of EOX (epirubicin, oxaliplatin, and capecitabine) appears similar to the chemotherapeutic regimens mentioned previously [10]. This form of therapy is clinically more applicable since it does not require central venous access for continuous administration. Furthermore, in the quest for the optimal treatment regimen, the FLOT4 multicentre randomised control trial reported superior results vis-à-vis OS to previous treatment regimens [11].

### Research aims

While perioperative chemotherapy for GC has become standard practice based on European guidelines, there remains a notable scarcity of real-world data comparing the outcomes of perioperative EOX therapy versus upfront surgery alone. The following key questions remain: Can this treatment be universally applied? Is it feasible and what are the immediate outcomes? Does it enhance surgical results, and are there associated risks? Is it universally beneficial? In this study, we aimed to investigate the safety and the short- and long-term advantages of perioperative chemotherapy in patients undergoing EOX treatment in a clinical setting.

## Materials and methods

### Study design and patient selection

This retrospective study was conducted at Helsinki University Hospital (HUH), a teaching institution that provides secondary and tertiary care for gastric cancer (GC) surgery, serving a population of around 2.2 million. Patients were identified through the electronic operating room database using the International Classification of Diseases (ICD- 10) code C16, corresponding to malignant stomach neoplasm, and various procedure codes related to gastrectomy (JCD10, JDA96, JDC00, JDC10, JDC20, JDC30, JDC96, JDD00, and JDD96). The data search was restricted to the period between January 1, 2006, and December 31, 2017.

### Data collection

We manually gathered data from patient records, including demographic information, American Society of Anaesthesiologists (ASA) classification, medications, comorbidities based on the Charlson Comorbidity Index [12], laboratory results, surgical details, length of hospital stay, admissions to the intensive care unit (ICU), reoperations, and readmissions occurring within 30 days post-discharge. Complications were classified according to Clavien–Dindo criteria [13] and the total burden of complications was assessed using the comprehensive complication index (CCI) [14]. Additionally, the date of diagnosis, typically established during gastroscopy, was recorded. Missing laboratory values are reported as missing to maintain transparency in the data collection process.

### Oncological and staging data

Oncological data were obtained from patient records, including information on possible pre- and postoperative EOX (epirubicin, oxaliplatin, and capesitabine) therapy and the number of treatment cycles administered. Preoperative staging was performed based on the CT report and confirmation of the CT findings. In the case of a discrepancy between the report and CT findings, two experienced surgeons re-evaluated them. In some cases, the preoperative staging also included an endoscopic view or an ultrasound. Patients were categorised either as having cT1–T2 or cT3–T4 tumours and metastatic lymph node positive (cN +) or negative (cN-) according to the preoperative CT findings. The tumour (cT) and nodal (cN) status were evaluated before the initiation of any EOX treatment to ensure accurate pre-treatment staging. For preoperative staging variables, cT and cN values were recorded as “undetermined” when they were not available due to the absence of preoperative imaging or insufficient staging information. As this retrospective study lacks explicit documentation for treatment choices, decisions regarding the assignment to perioperative EOX therapy or upfront surgery were made collaboratively by the surgeon and the patient, based on tumour stage, patient fitness, and chemotherapy availability.

### Pathological data

The pathological report determined the histology according to the Lauren classification [15] and staging, consistent with the AJCC TNM 7 th edition [16]. Postoperative mortality was defined as death within 30 days following gastrectomy. Follow-up data were manually collected from hospital patient records or the Population Information System. The Population Information System is an up-to-date service offered by the Finnish government (Digital and Population Data Services Agency) that maintains trustworthy records for the population, providing information on whether a person is living or deceased [17]. The last recorded date for the patient (defined as their most recent contact with a healthcare facility or hospital), any potential recurrence, long-term mortality, or cause of death was retrieved from the hospital's patient records. If follow-up was not provided at HUH, the patient records were obtained from the referral hospital to access follow-up data.

### Patient grouping

Patients were divided into two groups depending on whether they received preoperative EOX therapy. Neoadjuvant EOX therapy was typically offered to patients with locally advanced gastric cancer (e.g., cT3–T4 and/or cN +), as determined by preoperative staging. Initially, younger and fitter patients were prioritised, reflecting a cautious adoption of this regimen after its introduction in 2006. By 2010, EOX became more widely used as the standard treatment for locally advanced gastric cancer. Patients who received another form of preoperative oncological treatment were excluded from the study. Curative intent was defined as the aim of an R0 resection assessed by the surgeon intraoperatively, while R0 resection was defined as the complete removal of all cancerous tissue with clear microscopic margins, typically confirmed through examination of frozen sections taken from resection margins during surgery. Patients with limited resectable metastatic disease, such as a solitary lesion (e.g., ovarian metastasis), were included if an R0 resection was achieved. However, patients with unresectable or widespread metastatic disease were excluded from the analysis. Recurrence was identified by detection through imaging (commonly CT), surgery, or endoscopy. At our institution, follow-up imaging is only performed if symptoms or recurrence are suspected (e.g., elevated CEA or CA19 - 9 levels).

### Propensity score matching

Propensity score matching (PSM) utilised clinically relevant variables known to influence patient selection. These variables consisted of age, sex, cT, cN, and the Charlson comorbidity index. Additionally, the type of surgical resection (total or subtotal gastrectomy), extent of lymph node dissection (D0, D1, D1 +, and D2) and histological type (Lauren classification) were considered.

Match criterion was set to ± 0.25 standard deviations (SDs) of the logit of the propensity score. At a 1:1 ratio, we matched patients using the ‘nearest neighbour’ method. The effectiveness of matching was assessed by comparing the standardised mean difference (SMD) before and after matching based on pre- and intraoperative variables.

### Statistical analysis

All statistical analyses were performed using SPSS® version 29 (IBM, Armonk, New York, USA) or R [R Core Team (2022). R: A language and environment for statistical computing. R Foundation for Statistical Computing, Vienna, Austria, https://www.R-project.org/]. We analysed continuous variables with the student’s t-test (normally distributed) or the Mann–Whitney U-test (non-normally distributed). We report normally distributed continuous variables as the mean (± SD) and non-normally distributed variables as the median [interquartile range (IQR)]. We analysed categorical variables using the chi-square or Fisher’s exact test (if the number of events per cell < 5). Survival was estimated using the Kaplan–Meier method, and groups were compared using the log-rank test. We calculated OS from the date of diagnosis to the date of death for any reason or censored at the last date of follow-up if the patient remained living. Disease-specific survival (DSS) was calculated from the date of diagnosis to the date of death caused by GC or censored at the last date of follow-up if living or as the date of death for any other reason. Disease-free survival (DFS) was calculated from the date of diagnosis to the date of recurrence or death from GC, whichever occurred first or censored at the date of last follow-up if living without recurrence, or death for reason other than GC. Cox regression was used in the matched cohort to adjust for the effect of adjuvant therapy for OS and DSS, results are presented as hazard ratios (HR). All tests were two-sided, and we considered *P* < 0.05 as statistically significant.

## Results

### Patient cohort

**Fig. 1:**
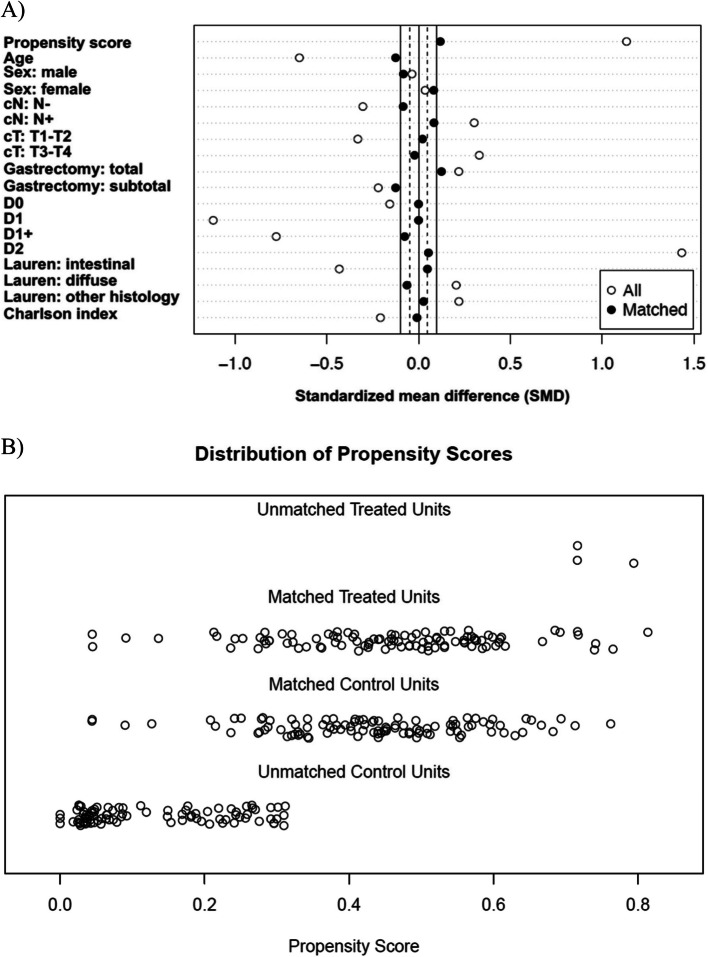
Propensity score-matching utilising clinically relevant variables (**A**) and distribution of propensity scores (**B**)

Between 1 January 2006 and 31 December 2017, a total of 310 patients underwent elective gastrectomy with a curative intent for GC. Among them, 105 received preoperative EOX treatment and 205 underwent US. After PSM, 102 patients remained in each group and were well balanced on basic characteristics (Fig. 1 and Table 1).

**Table 1:** Baseline characteristics of propensity score–matched patients

	EOX group (*n* = 102)	US group (*n* = 102)	*P*
Median age, in years (IQR)	65.6 (60.6–70.2)	66.3 (57.3–73.7)	0.379
Sex, male (%)	54 (52.9)	54 (52.9)	1.000
Mean body mass index, in kg/m^2^ (± SD)	26.3 (4.3)	25.3 (4.5)	0.102
ASA physical status, n (%)			0.330
1	5 (4.9)	10 (9.8)	
2	34 (33.3)	36 (35.3)	
3	53 (52.0)	51 (50.0)	
4	10 (9.8)	5 (4.9)	
Comorbidities, n (%)			
Myocardial infarction	5 (4.9)	8 (7.8)	0.390
Congestive heart failure	3 (2.9)	4 (3.9)	1.000
Peripheral vascular disease	4 (3.9)	2 (2.0)	0.683
Cerebrovascular disease	1 (1.0)	2 (2.0)	1.000
Hemiplegia	1 (1.0)	2 (2.0)	1.000
COPD or asthma	10 (9.8)	7 (6.9)	0.447
Connective tissue disease	2 (2.0)	1 (1.0)	1.000
Liver disease			
Mild	2 (2.0)	1 (1.0)	1.000
Diabetes mellitus			
without complications	15 (14.9)	13 (12.7)	0.663
with complications	2 (2.0)	0	0.246
Kidney disease (moderate/severe)	2 (2.0)	6 (5.9)	0.279
Cancer (metastatic)	5 (4.9)	7 (6.9)	0.552
Peptic ulcer	12 (11.8)	6 (5.9)	0.139
Charlson comorbidity index, n (%)			
mild (0-2)	58 (56.9)	60 (58.8)	0.777
moderate (3-4)	34 (33.3)	30 (29.4)	0.546
Severe (≥ 5)	10 (9.8)	12 (11.8)	0.652
Mean Charlson comorbidity index (± SD)	2.8 (1.2)	2.9 (1.4)	0.870
Medication, n (%)			
Anticoagulant	8 (7.8)	2 (2.0)	0.052
Corticosteroid	6 (5.9)	2 (2.0)	0.279
Immunosuppressive	1 (1.0)	0	1.000
Laboratory, median (IQR)			
CA19 - 9	8.0 (3.0–15.8)	8 (3.0–15.0)	0.840
Missing no	2	25	
CEA	2.3 (1.4–3.4)	1.9 (1.3–3.1)	0.168
Missing no	0	5	
Hb	120.5 (110.0–131.3)	132 (118.0–139.0)	< 0.001
Missing no	0	1	
Alb	37.3 (34.5–39.8)	37.8 (34.0–40.2)	0.599
Missing no	0	3	
Creatinine	67.5 (57.0–78.3)	70.0 (60.5–79.0)	0.257
Missing no	0	1	
Preoperative cT (%)			
cT1 - 2	36 (35.6)	38 (37.6)	0.622
cT3 - 4	65 (64.4)	61 (60.4)	
cT undetermined	1 (1.0)	3 (2.0)	
Preoperative cN (%)			
cN negative	61 (59.8)	63 (61.8)	0.942
cN positive	40 (39.2)	38 (37.3)	
cN undetermined	1 (1.0)	1 (1.0)	

*Abbreviations*: *EOX *epirubicin, oxaliplatin, and capesitabine, *US *upfront surgery, *IQR *interquartile range, *SD *standard deviation, *ASA *American Society of Anesthesiologists class, *COPD *chronic obstructive pulmonary disease, *Hb *haemoglobin, *Alb *albumin

### EOX treatment completion

In the EOX group, 89 patients (87.3%) completed three or more cycles of preoperative EOX treatment, while 5 patients (4.9%) received two cycles, and 8 patients (7.8%) received only one cycle of EOX. Preoperative EOX therapy was terminated in a small subset of patients due to poor blood values (1 patient), clinical deterioration (4 patients), diarrhoea (1 patient), and other reasons (8 patients). A total of 65 patients (63.7%) received postoperative adjuvant chemotherapy in the EOX group compared to 50 patients (49.0%) in the US group (*P* = 0.048). In the EOX group, 41 patients (40.2%) received three or more cycles of EOX, 8 (7.8%) two cycles, 5 (4.9%) one cycle, and another 11 patients (10.7%) received a different postoperative treatment regimen. The reasons for incomplete postoperative chemotherapy in the EOX group included postoperative complications in 7 cases, a deteriorating physical condition in 8 cases, noncompliance in 7 cases, and various other reasons for the remaining 16 cases.

### Patient characteristics and operative details

Patients in the US group had higher preoperative haemoglobin levels. Other basic characteristics were comparable between groups. No significant differences were observed with respect to the type of resection and lymphadenectomy, surgical approach or reconstruction techniques (Table 2). Splenectomy was performed in 24 patients (23.5%) in the EOX group and 41 patients (40.2%) in the US group, with the splenectomy rate being significantly higher in the US group (*P* = 0.011). Among these, 31 patients (47.4%) had T4 tumours, including 10 patients in the EOX group and 21 patients in the US group. A sub-analysis comparing splenectomised T4 patients between the two groups revealed no statistically significant difference (*P* = 0.457). Other organ resections were comparable between the two groups. Intraoperative blood loss did not differ between groups, but the operative time (*P* = 0.002) was statistically significantly longer in the EOX group (Table 2).

**Table 2:** Operative details of propensity score–matched patients

	EOX group (*n* = 102)	US group (*n* = 102)	*P*
Type of resection, n (%)			0.574
Total gastrectomy	58 (56.9)	54 (52.9)	
Distal gastrectomy	44 (43.1)	48 (47.1)	
Approach, n (%)			1.000
Open	98 (97.1)	99 (96.1)	
Laparoscopic	4 (3.9)	3 (2.9)	
Lymph node dissection, n (%)			1.000
D1	2 (2.0)	1 (1.0)	
D1 +	2 (2.0)	3 (2.9)	
D2	98 (96.1)	98 (96.1)	
Time median, in minutes (IQR)	222.5 (192.3–269.8)	206.0 (173.0–226.8)	0.002
Blood loss (ml), median (IQR)	600.0 (400.0–1000.0)	500.0 (300.0–900.0)	0.132
Resection of adjacent or another organ			
Splenectomy	24 (23.5)	41 (40.2)	0.011
Liver	1 (1.0)	0	1.000
Pancreas	4 (3.9)	6 (5.9)	0.517
Other^a^	6 (5.9)	7 (6.9)	0.774
Reconstruction, n (%)			0.250
Roux-en-Y	63 (61.8)	58 (56.9)	
Billroth I	0	3 (2.9)	
Billroth II	39 (38.2)	41 (40.2)	

*Abbreviations: EOX *epirubicin, oxaliplatin, and capesitabine, *US *upfront surgery, *IQR *interquartile range

^a^This included the following resections: 3 transverse colon resections, 1 rectum resection, 2 bowel resections, 2 adrenal gland resections, 1 kidney resection, 1 breast resection, and 3 ovary resections

### Postoperative complications

The morbidity rates were comparable between both groups. The rate of severe complications (Clavien–Dindo grades III, IV, and V) was 24.5% in the EOX group and 17.6% in the US group, but we observed no difference in the CCI. The ICU admission rate was higher in the EOX group (16.7%) compared to the US group (6.9%**,** *P* = 0.030). Among ICU-admitted patients, the median intraoperative blood loss was 950 mL (IQR: 455–1375), substantially higher than the overall median blood loss in the EOX (500 mL) and US (600 mL) groups, though this difference was not statistically significant (*P* = 0.924). ASA Class distributions indicated more patients with ASA IV in the EOX group (7 vs. 1 in the US group, *P* = 0.362), and Charlson Comorbidity Index scores ≥ 3 were more common in the EOX group (11 vs. 4, *P* = 0.570). The median operative time for ICU patients was 228 min (IQR: 198–323), with no significant difference between groups (*P* = 0.116). Additional organ resections were more frequent in ICU admitted patients in the EOX group. This included one liver resection, one pancreatic resection, three other resections and five splenectomies compared to only two splenectomies in the US group. Furthermore, the days spent in the ICU, the length of stay in the hospital, reoperation rate, and readmission rate were comparable between groups. Postoperative mortality was 1.0% for the EOX group and 2.0% for the US group (Table 3).

**Table 3:** Operative results of propensity score–matched patients

	EOX group (*n* = 102)	US group (*n* = 102)	*P*
Clavien–Dindo grade, n (%)^a^			0.877
0	12 (11.8)	13 (12.7)	
1	24 (23.5)	31 (30.4)	
2	40 (39.2)	41 (40.2)	
3a	17 (16.7)	11 (10.8)	
3b	2 (2.0)	2 (2.0)	
4a	3 (2.9)	2 (2.0)	
4b	2 (2.0)	1 (1.0)	
5	1 (1.0)	2 (2.0)	
CCI, median (IQR)	22.6 (8.7–30.8)	22.6 (8.7–34.2)	0.959
Reoperation, n (%)	5 (4.9)	4 (3.9)	1.000
ICU admission, n (%)	17 (16.7)	7 (6.9)	0.030
ICU stay (days), median (IQR)^b^	3.0 (2.0–7.0)	3.0 (2.0–8.0)	0.871
Length of hospital stay (days), median (IQR)	8.0 (7.0–11.0)	9.0 (7.0–12.0)	0.258
Readmission, n (%)	11 (10.8)	9 (8.8)	0.638
30-day mortality, n (%)	1 (1.0)	2 (2.0)	1.000
90-day mortality, n (%)	1 (1.0)	3 (2.9)	0.621

*Abbreviations*: *EOX *epirubicin, oxaliplatin, and capesitabine, *US *upfront surgery, *IQR *interquartile range, *CCI *comprehensive complication index, *ICU *intensive care unit

^a^Highest complication grade

^b^For patients admitted to ICU

### Pathological details

We observed no differences between groups regarding histology, tumour location or the R class. In 9 cases (8.8%) from the EOX group, no tumour was found in the specimen, indicating a beneficial response to EOX treatment; in addition, no tumour was found in one case (1.0%) in the US group (*P* = 0.009). In the US group, pT4 tumours were more common. Otherwise, the depth of invasion was similar in both groups. The number of harvested lymph nodes or cM category did not differ between groups. Interestingly, the number of metastatic lymph nodes (*P* = 0.004) was significantly lower in the EOX group. In addition, the tumour size (*P* < 0.001) was also significantly smaller in the EOX group (Table 4).

**Table 4:** Pathological details of propensity score–matched patients

	EOX group (*n* = 102)	US group (*n* = 102)	*P*
Histology, n (%)			0.564
Intestinal	25 (24.5)	26 (25.5)	
Diffuse	62 (60.8)	66 (64.7)	
Mixed/other^a^	15 (14.7)	10 (9.8)	
Tumour location, n (%)			0.273
Lower (antrum, angulus)	36 (35.3)	43 (42.2)	
Middle (body)	60 (58.8)	49 (48.0)	
Upper (fundus, cardia)	6 (5.9)	8 (7.8)	
Other^b^	0	2 (2.0)	
pT category, n (%)			0.025
pT0^c^	9 (8.8)	1 (1.0)	
pT1	18 (17.6)	21 (20.6)	
pT2	22 (21.6)	17 (16.7)	
pT3	33 (32.4)	29 (28.4)	
pT4	20 (19.6)	34 (33.3)	
Lymph node status, n (%)			0.008
Negative	54 (54.9)	37 (36.3)	
Positive	46 (45.1)	65 (63.7)	
cM category, n (%)	5 (4.9)	6 (5.9)	0.757
Lymph node harvested, median (IQR)	21 (14-30)	24 (17–33)	0.293
Stage, n (%)			0.030
0	7 (6.9)	1 (1.0)	
IA	13 (12.7)	15 (14.7)	
IB	19 (18.6)	8 (7.8)	
IIA	13 (12.7)	16 (15.7)	
IIB	11 (10.8)	16 (15.7)	
IIIA	16 (15.7)	9 (8.8)	
IIIB	11 (10.8)	23 (22.5)	
IIIC	6 (5.9)	8 (7.8)	
IV	5 (4.9)	6 (5.9)	
Metastatic lymph nodes, median (IQR)	0 (0–4)	2 (0–6)	0.004
Tumour size (mm), median (IQR	30.0 (20.0–50.0)	50 (28.0–85.0)	< 0.001
R class, n (%)			1.000
R0	98 (96.1)	97 (95.1)	
R1	3 (2.9)	5 (4.9)	
R2	0	1 (1.0)	

*Abbreviations*: *EOX *epirubicin, oxaliplatin, and capesitabine, *US *upfront surgery, *IQR *interquartile range

^a^Histology is classed as ‘other’ when the histology could not be determined from the gastroscopy biopsies and the final specimen showed no tumour due to a total response in the EOX group

^b^Two cases in the US group had cancer in the anastomosis of the remnant ventricle due to a previous gastric resection of peptic ulcer disease

^c^Nine cases of a total response in the EOX group. One patient in the US group had GC in the gastroscopy biopsies, but this was not confirmed in the specimen by pathology

### Long-term survival

**Fig. 2:**
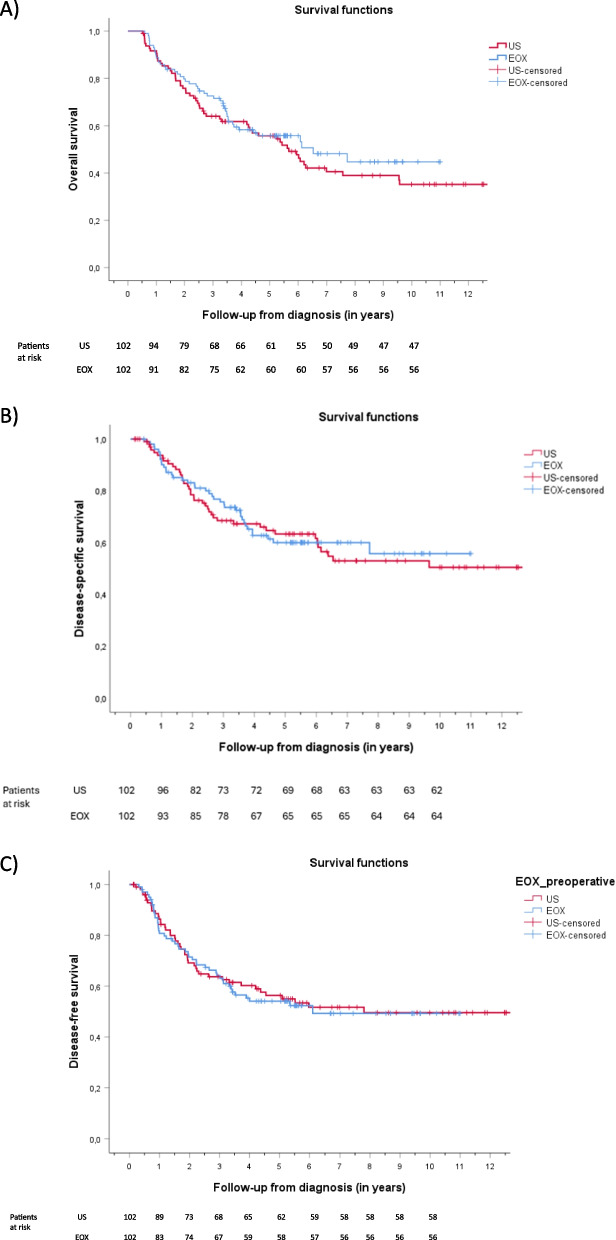
Overall survival (*P* = 0.276) (**A**), disease-specific survival (*P* = 0.779) (**B**), and disease-free survival (*P* = 0.861) (**C**) of propensity-score matched GC patients from diagnosis

OS, DSS, and DFS were similar between the EOX and US groups (Fig. 2). The median follow-up from diagnosis was 4.1 years (IQR 1.9–6.5) for all patients, 3.9 years (IQR 2.4–5.7) for the EOX group, and 4.3 years (IQR 1.7–7.4) for the US group (*P* = 0.723). The mean estimated OS from diagnosis was 7.0 years [95% confidence interval (CI) 6.1–7.9] for the EOX group and 7.6 years (95% CI 6.4–8.9) for the US group (*P* = 0.276). The mean estimated DSS from diagnosis was 7.4 years (95% CI 6.5–8.3) for the EOX group and 8.9 years (95% CI 7.7–10.2) for the US group (*P* = 0.779). The mean estimated DFS from diagnosis was 6.5 years (95% CI 5.6–7.5) for the EOX group and 8.4 years (95% CI 7.1–9.7) for the US group (*P* = 0.861).

According to Cox regression analysis in the matched cohort, in terms of OS adjuvant therapy alone (HR 0.95, 95% CI: 0.55–1.62, *p* = 0.842) or preoperative EOX alone (HR 1.25, 95% CI: 0.70–2.24, *P* = 0.450) were not associated with survival benefit. There was a trend for improved OS when both treatments were combined, but this was not statistically significant (HR 0.54, 95% CI: 0.24–1.22, *P* = 0.140).

For DSS, preoperative EOX alone was associated with an increased hazard of disease-related mortality (HR 1.99, 95% CI: 1.0004–3.9476, *P* = 0.05), although this result is borderline statistically significant. In contrast, adjuvant therapy alone did not significantly affect DSS (HR 1.65, 95% CI: 0.87–3.10, *P* = 0.123). The interaction between treatments demonstrated a strong protective effect (HR 0.31, 95% CI: 0.12–0.75, *P* = 0.010), suggesting that patients who received both preoperative EOX and adjuvant therapy had significantly improved survival compared to those receiving either treatment alone (Table 5).

**Table 5:** Effects of neoadjuvant and adjuvant treatment on OS and DSS: A Cox regression analysis in a matched cohort of 204 GC patients

Covariates	Coefficient	HR (95% CI)	*P*
*Overall survival*			
Adjuvant treatment	− 0.054	0.95 (0.55–1.62)	0.842
Preoperative EOX	0.225	1.25 (0.70–2.24)	0.450
Interaction term^a ^(Adjuvant treatment × Preoperative EOX)	− 0.608	0.54 (0.24–1.22)	0.140
*Disease-specific survival*			
Adjuvant treatment	0.498	1.65 (0.87–3.10)	0.123
Preoperative EOX^b^	0.687	1.99 (1.00–3.95)	0.05
Interaction term^a^ (Adjuvant treatment × Preoperative EOX)	− 1.19	0.31 (0.12–0.75)	0.010

^a^Interaction terms represent the combined effect of adjuvant treatment and preoperative EOX

^b^The hazard ratio for preoperative EOX in DSS is 1.99, with a 95% CI of 1.0004–3.9476 and an exact p-value of 0.050000000000000044 (reported as 0.05 in the table). This value lies precisely at the conventional threshold for significance, indicating a borderline result that should be interpreted with caution

## Discussion

### Overview of findings

This is one of the few European studies to compare the outcomes of perioperative treatment among GC patients. Our findings suggest that perioperative chemotherapy is safe vis-à-vis postoperative mortality and morbidity. Such treatment may reduce tumour size, the number of metastatic lymph nodes, and, in some cases, lead to a total response. We observed no difference in OS, DSS or DFS between the EOX group or US group.

### Comparison with long-term outcomes

Compared with long-term outcomes in East Asia, survival remains poor for GC patients in Europe. The reason for this difference remains unknown but is likely multifactorial. A lack of screening programmes, clinicopathological features in GC, and the extent of lymphadenectomy may offer some explanation. It is quite clear, however, that neoadjuvant chemotherapy is of interest. First, patient tolerability is expected to be higher before versus after surgery. Second, downstaging disease before surgery offers the possibility of a total response and increasing the possibility of achieving R0 resections. Third, we may obtain valuable information on postoperative treatment regimens. And fourth, eliminating possible micrometastasis at an earlier stage may improve longer term survival. Two European trials [8, 9] led to changes in Western treatment guidelines by demonstrating the benefits of perioperative chemotherapy in improving surgical outcomes and OS rates.

### Safety and efficacy of perioperative treatment

According to our findings, perioperative treatment is safe and does not increase the risk of complications or mortality. This agrees with the two previous studies [8, 9]. We found that patients who received perioperative chemotherapy showed markedly lower haemoglobin levels, possibly resulting from the toxic effects of EOX therapy. A splenectomy was more common in the US group, with the majority of these procedures performed during the early stages of our study. This may partly reflect the presence of T4 tumours, which often require more extensive resection, as well as the historically more radical surgical approach. Recently, spleen-preserving procedures have become more common [18, 19]. ICU-admissions were more frequent in the EOX group, likely due to multifactorial reasons. Both groups of ICU-admitted patients experienced higher blood loss compared to their respective overall groups, suggesting that intraoperative bleeding may have contributed to ICU management decisions. However, the higher prevalence of comorbidities (Charlson Comorbidity Index scores ≥ 3), a greater number of ASA IV patients, and the increased frequency of adjacent or other organ resections in the EOX group appear to be the primary factors driving the higher ICU admission rate.

### Pathological findings and long-term outcomes

Mirroring the results of the MAGIC trial, neoadjuvant treatment was implemented at a high compliance rate, with 87.3% receiving three or more treatment cycles. Moreover, 40.2% received the planned therapeutic regimen of three or more cycles, 12.7% received two or less cycles postoperatively, and 10.7% received another therapeutic regimen compared with 49.5% in the MAGIC trial. Our pathological findings match previous studies, whereby we detected a significant reduction in the tumour size and the number of metastatic lymph nodes [8]. Notably, 9 cases (8.8%) were classified as total responders in the EOX group, identifying a trend towards downstaging. However, the rate of R0 resection did not differ between groups.

Long-term outcomes, including overall and disease-free survival, were similar between the groups, and we did not observe an overall survival benefit with perioperative therapy. However, our multivariable analysis revealed a significant improvement of DSS among patients who received pre and postoperative therapy according to the treatment protocol. This finding might be biased as only the fit patients will continue receiving chemotherapy after surgery, and patients with poor fitness, disease progression, or with complications will drop out from the treatment after surgery. This differs from the findings from previous studies, even when adjusted for propensity score matching [20]. Some of the differences can be explained by clinicopathological and methodological issues. The MAGIC trial did not categorise the tumours based on histology, tumour size was not adequately reported, and the T stage was much lower compared with our study. In our population, only 5.9% of the tumours affected the upper part of the stomach and no tumours were categorised as oesophageal in our patient cohort. In the MAGIC trial, 25% of cases were either lower oesophageal or gastroesophageal junction tumours. In the French ACCORD- 07 trial, 75% of cases were oesophageal or esophagogastric tumours. Previous research has demonstrated that cancers in the upper part of the stomach and oesophagus are more sensitive to chemotherapy [21]. Histology may be crucial: 60.8% in the EOX group patients had diffuse type GC, which associates with a poor response to chemotherapy [22]. Finally, the surgery conducted in the MAGIC trial did not meet current standards, with only 42.5% of patients undergoing a D2 lymphadenectomy. Moreover, 69.3% of the procedures were considered curative, which may contribute to the differences in survival. In our study, all patients were subjected to surgery with a curative intent and 96.1% underwent a D2 lymphadenectomy.

### Study limitations and strengths

In this single-centre study, the main strength lies in its reliable long-term follow-up period, collected over a 12-year period which included consecutive patients undergoing gastrectomy with a curative intent for GC. Notably, the study did not rely on registries; instead, all patient records underwent manual screening and data collection, enhancing the data quality, which is both a limitation and a strength.

However, inherent limitations exist due to the retrospective design of our study, including biases such as patient selection and potential disparities in prognostic factors. Despite utilising PSM to address these issues, unmeasured or unknown confounding variables may persist. While detailed toxicity data were not systematically collected, we provide information on treatment discontinuation rates and reasons, offering some insight into the tolerability of EOX therapy. Patients referred for EOX but who did not undergo curative surgery were excluded, limiting insights into treatment feasibility for all eligible patients. During the study period (2006–2017), staging laparoscopy was not routinely performed at our institution due to resource constraints. Since 2019, it has been routinely adopted for high-risk patients, such as those with suspected peritoneal dissemination. This evolving practice should be considered when interpreting the staging and treatment outcomes reported in this study. Additionally, the sample size is small, largely due to the low incidence of GC in Finland, which averages just under 700 cases annually. Nevertheless, this limitation is common among Western series due to the rarity of GC. In addition, a reliance on FLOT (fluorouracil, leucovorin, oxaliplatin, and docetaxel) treatment has replaced EOX treatment in many cases at our hospital. It is important to note that during the study period (2006–2017), EOX was the standard perioperative chemotherapy regimen at our institution. At that time, newer regimens, such as FLOT, were not yet adopted into routine clinical practice. The results of our study should thus be interpreted within the context of available treatments during the study timeframe, acknowledging that FLOT has since demonstrated superior outcomes in subsequent trials and is now the preferred regimen in many institutions.

## Conclusions

To conclude, our results demonstrate that perioperative EOX therapy in GC patients is feasible, with low postoperative morbidity and mortality rates. The evidence suggests that neoadjuvant treatment may decrease the tumour size and reduce the number of metastatic lymph nodes. In 8.8% of cases, we detected a complete response indicating that a certain subgroup of patients can benefit from the treatment. Exploring the reasons for this finding remain of great interest for the future. However, we found no long-term benefit of treatment when comparing treatment groups. Further prospective, randomised studies are required to explore treatment feasibility, toxicity, and to identify subgroups that may benefit most from perioperative chemotherapy in GC patients.

## Supplementary Information

Additional file 1.

## Data Availability

The data that support the findings of this study are not openly available due to reasons of sensitivity and are available from the corresponding author upon reasonable request. Data are located in controlled access data storage at Department of Abdominal Surgery, Helsinki University Hospital.

## Abbreviations

GC: Gastric Cancer
US: Upfront surgery
EOX: Epirubicin, oxaliplatin, capecitabine
PSM: Propensity score matching
CCI: Comprehensive complication index
OS: Overall survival
DSS: Disease-specific survival
DFS: Disease-free survival
ICU: Intensive care unit
ECF: Epirubicin, cisplatin, and 5-fluorouracil
HUH: Helsinki University Hospital
ASA: American Society of Anesthesiologists
cN +: Lymph node positive
cN-: Lymph node negative
AJCC TNM: American Joint Committee on Cancer classification of malignant tumours
CEA: Carcinoembryonic antigen
CA19 - 9: Cancer antigen 19–9
SD: Standard deviations
SMD: Standardised mean difference
IQR: Interquartile range
FLOT: Fluorouracil, leucovorin, oxaliplatin, and docetaxel
