# Supplementary figures and images for: Short- and long-term outcomes after perioperative EOX therapy versus upfront surgery for gastric cancer: a single-centre propensity score–matched cohort study
Source: BMC Surg. 2025 Apr 28;25:184. doi: 10.1186/s12893-025-02919-4 (PMC12039105; doi:10.1186/s12893-025-02919-4)

Supplementary figure 1. Flow-chart of patient selection


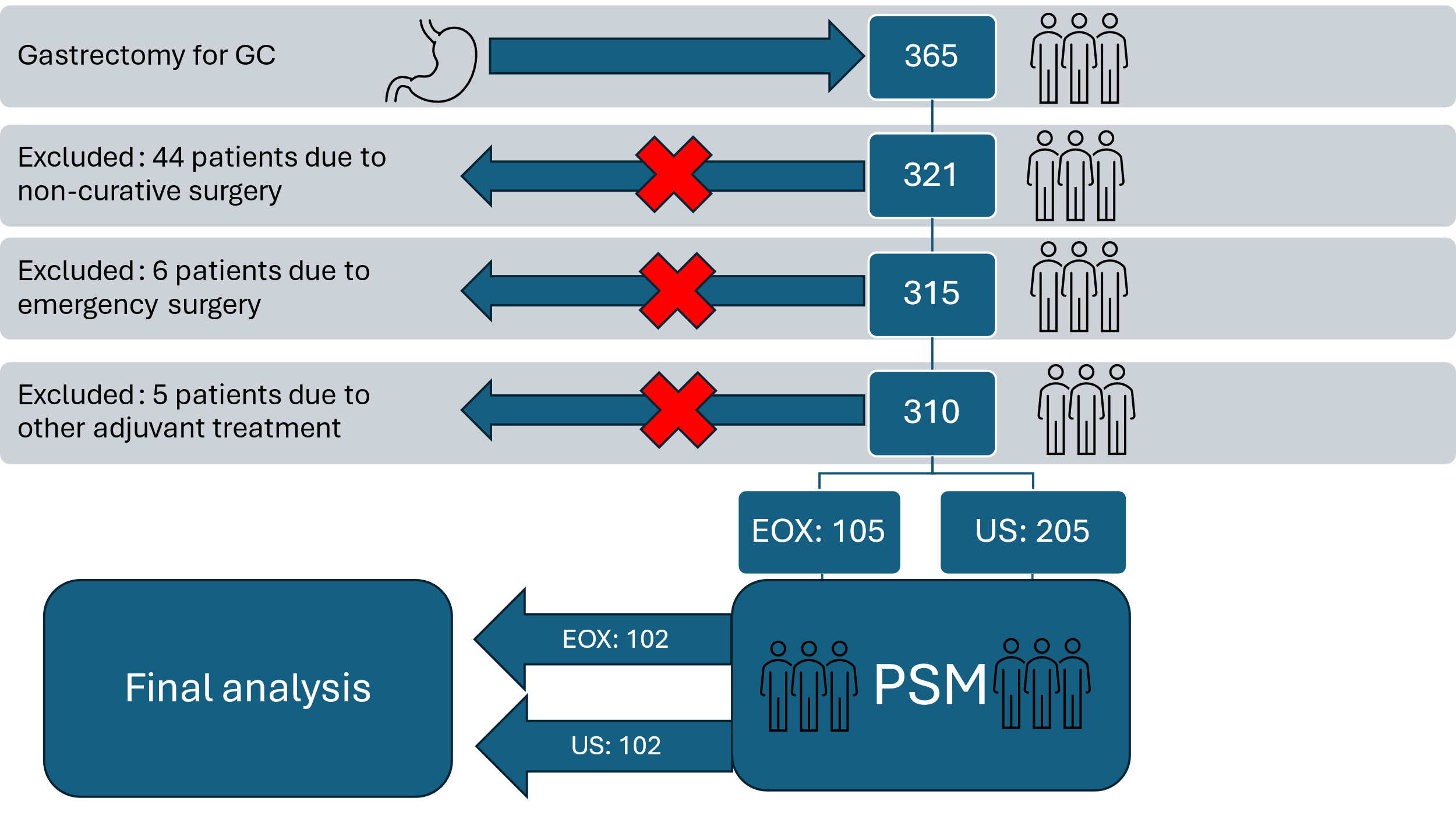

Supplement: Supplementary file 1 — Additional file 1. [file 12893_2025_2919_MOESM1_ESM.docx]
